# Traumatic Brain Injury (TBI) Detection: Past, Present, and Future

**DOI:** 10.3390/biomedicines10102472

**Published:** 2022-10-03

**Authors:** Ali T. Alouani, Tarek Elfouly

**Affiliations:** Electrical and Computer Engineering Department, College of Engineering, Tennessee Technological University, Cookeville, TN 38505, USA

**Keywords:** traumatic brain injury, neuroimaging, electroencephalography, preprocessing, artifacts removal, detection, artificial intelligence

## Abstract

Traumatic brain injury (TBI) can produce temporary biochemical imbalance due to leaks through cell membranes or disruption of the axoplasmic flow due to the misalignment of intracellular neurofilaments. If untreated, TBI can lead to Alzheimer’s, Parkinson’s, or total disability. Mild TBI (mTBI) accounts for about about 90 percent of all TBI cases. The detection of TBI as soon as it happens is crucial for successful treatment management. Neuroimaging-based tests provide only a structural and functional mapping of the brain with poor temporal resolution. Such tests may not detect mTBI. On the other hand, the electroencephalogram (EEG) provides good spatial resolution and excellent temporal resolution of the brain activities beside its portability and low cost. The objective of this paper is to provide clinicians and scientists with a one-stop source of information to quickly learn about the different technologies used for TBI detection, their advantages and limitations. Our research led us to conclude that even though EEG-based TBI detection is potentially a powerful technology, it is currently not able to detect the presence of a mTBI with high confidence. The focus of the paper is to review existing approaches and provide the reason for the unsuccessful state of EEG-based detection of mTBI.

## 1. Introduction

Traumatic brain injury can lead to three types of injuries: a collection of the blood within the skull called intracranial hematoma (ICH) [1], an elevated intracranial pressure (ICP) [2], and a midline shift (MLS) [3]. Nonfatal injuries can result in a severe lifetime disability, which has significant impact on the injured and his/her family, as well as on healthcare cost. The estimated annual worldwide number of TBI cases is 69 millions [4]. In 2016, the annual total cost of nonfatal TBI was about 40.6 billion dollars in the United States [5]. For the armed forces in the US, it was found that the TBI cases were 91 percent higher for deployed combat forces than those who are not in a combat zone [6]. It is known that the sooner nonfatal TBI is detected and treated, the lesser the long term impact on the injured will be. The first hour following a brain injury is known as the golden hour [7,8,9]. The Glasgow Coma Scale (GCS) is used to classify the severity of TBI injury as mild, moderate and severe according to their level and severity [10]. Mild TBI (mTBI) accounts for 70 to 90 percent of all TBI cases [11]. In the case where mTBI is not diagnosed, its effect may lead to limited to impaired cognitive function, fatigue, depression, irritability, and headaches. This paper provides an assessment of the techniques used in TBI detection and suggests important issues that need to be addressed in order for EEG-based TBI detection technology to become accurate and more acceptable to practicing medical professionals. This paper is organized as follows. Section 2 provides a brief historical review of TBI detection. Section 3 contains an overview of TBI detection using conventional methods along with the limitations of this approach. Section 4 focuses on the use of artificial intelligence for TBI detection. Section 5 discusses the limitations of the current state of the art and proposes future research directions to improve the effectiveness of TBI detection using EEG. Section 6 contains conclusions and discussions.

## 2. Brief Historical Review

An ionic current flows through the brain’s neurons and creates oscillating electrical voltage. In 1924, the German psychiatrist Hans Berger recorded such fluctuations known as the electroencephalogram (EEG) and observed that different locations of the brain emit different waves. EEG was the main neurologic and psychiatric diagnostic tool for the period of 1930 to 1970 until the discovery of computed tomography (CT) in 1970 and magnetic resonance imaging (MRI) in 1980 [12].

The first clinical CT scan was performed in 1971. The current practice for diagnosing TBI uses neuroimaging and clinical examination. A survey of a rare condition of TBI known as traumatic internal carotid artery dissection (TICAD) was provided in [13]. The survey reveals that all the clinical studies used a small number of patients, where either one or a combination of CT, MRI and magnetic resonance angiography (MRA) were used. It was also concluded that there was no universal diagnosis technique to detect TICAD. A comprehensive survey of CT-based automated TBI detection was published in 2021 [14]. CT can only capture momentary snapshots of the dynamically evolving process of TBI. It can detect intracranial in head injuries, but cannot be used to exclude the presence of TBI. On the other hand, fMRI allows for functional brain mapping with high spatial resolution. This is important in pinpointing the location of the injury whenever it is detected. Unfortunately, because of its poor time resolution, fMRI may not be able to detect fast changing events as a result of neurochemical and neurometabolic activities immediately after brain injury. This is the case for other advanced imaging techniques. The study conducted in [15] raised questions about the credibility of the clinical use of neuroimaging for mTBI detection. Furthermore, clinical neuroimaging systems are expensive and bulky and not portable. They may not be suitable for use as the preliminary diagnosis tools in emergency cases when the patient is away from an emergency room or an imaging center. This is the case of a brain-injured football player, a person injured in a car accident, or a soldier in the field as a result of a sizeable explosion nearby or a gun shot. In the last two decades, the affordability of computing power, invention of digital recording and low noise electronics/sensors led to high temporal, as well as spatial, resolution EEG tests. These facts open up the door for EEG as a potential low cost and portable alternative to neuroimaging techniques as far as brain mapping [16]. EEG is currently used in many applications, including medical [17], emotion recognition [18], computer–brain interface (CBI) [19], and neuromarketing [20]. Conceivably, an EEG system may be used either alone or in combination with neuroimaging tests to provide a high level of accuracy in detecting TBI. Unfortunately EEG is corrupted with environmental noise and artifacts. The challenge is how to use it successfully to provide accurate and reliable TBI detection with a minimum false detection rate.

## 3. Conventional TBI Detection Using EEG

EEG signals are voltage signals collected with respect to a neutral reference electrode(s). Even though EEG contains valuable information about the brain waves that can be used for TBI detection, the brain waves amplitudes are very small, typically less than 100 µV. Furthermore, the recordings themselves are distorted by physical and non-physiological noises called artifacts. Non-physiological artifacts include electrode displacement, environment, and electrode-scalp impedance. Physiological artifacts include the effect of eye movement, blinking, muscle activity and cardiac activity. Such artifacts, if not removed, can lead to misleading TBI detection. Before any automated TBI detection, EEG signals must be first preprocessed to remove noise and artifacts.

### 3.1. EEG Signal Preprocessing

A significant amount of research work has been conducted to remove noise and artifacts form EEG signals. Artifacts removal uses linear regression, filtering/regression, independent component analysis (ICA), and principal component analysis (PCA) or a combination of different techniques [21,22,23,24,25,26,27,28]. Currently, for regression or blind source separation, it is assumed that the EEG model is linear, whereas the noise that models the artifacts is additive. The brain waves are also assumed to be stationary. The use of principal component analysis is based on the assumption that EEG signals and the artifacts are independent and linearly mixed with the true brain wave signals. The principal component analysis (PCA) uses orthogonal transformation under the assumption that neuronal activity waves are orthogonal to artifacts. Most of the studies focus on the removal of a particular artifact. There is no known solution that accommodates the different types of artifacts at once. Brain wave signals are non-stationary [29], non causal and nonlinear. Obtaining the average of the collected EEG signal over a relatively long period of time, in order to get around the non-stationarity of the signal, will make it less sensitive to the fast dynamics of the cortex that requires sampling in the order of milliseconds. The current state of the art in EEG preprocessing using analytical tools is expected to have limited performance with potential false alarm in detection.

### 3.2. TBI Detection Using EEG

The physiological method of evaluating the TBI level of injury is the Glasgow coma scale (GCS) obtained by adding the scores from eye opening, verbal response and motor response. The visual inspection of EEG may be successfully performed by a highly trained professional. Unfortunately, those professionals are in short supply. Furthermore, when it comes to quantifying the oscillatory activities of the brain waves, visual inspection sometimes fails to even differentiate between normal and abnormal brain waves. In an attempt to help the detection process, the concept of quantitative EEG (qEEG) has been introduced. Quantitative EEG represents a set of features extracted from the EEG signals to assess the functional state of the brain. The frequency bands of clinical interest in which brain waves oscillate are delta (0.5–4 Hz), theta (4–7 Hz), alpha (8–13 Hz), beta (14–30 Hz), and gamma (30–100 Hz) [30]. The features extraction uses signal-processing-derived tools, such as signal powers, power spectrum parameters, regularity measures, and coherence [31,32,33,34]. Several features can be extracted from the EEG signals. These include relative power, amplitude symmetry, coherence and phase difference [35]. Given the qEEG feature, multivariate analysis and discriminant functions are used to detect the existence of a TBI and its severity [33,35,36,37,38,39,40]. There is no universal index for TBI detection at this time. As pointed out earlier, due to the nonstationary nature of EEG signals and the fact that the signal features can vary for the same person and from person to person, the quantitative analysis using qEEG for TBI detection is helpful, but not reliable. There is no guarantee that the extracted features will have a unique behavior for TBI. At this time, there is no universal clinical index for TBI detection. It is very difficult to establish the one-to-one mapping between the TBI severity index and the qEEGs.

If one treats the TBI severity index as the output of an unknown complex map whose inputs are the EEG signals, one can think of the artificial intelligence to help establish such a map. Recent advances in artificial intelligence and especially machine learning and deep learning led to successfully obtaining the proper output, given the input information, without knowing the actual analytical map between inputs and outputs. In what follows, the paper will focus on EEG-based TBI detection using artificial intelligence.

## 4. TBI Detection Using Artificial Intelligence

**Figure 1:**
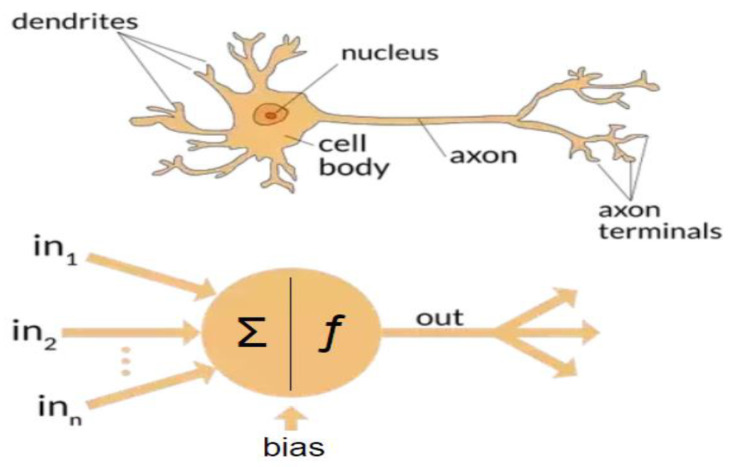
Biological and artificial neuron.

When it comes to learning and making complex decisions, even when based on partial information, the human brain does miraculous things. The human brain is equipped with approximately 100 billion neurons connected by about 1000 trillion synapses. Human learning takes place via adjustment of the synapses strength during training and generalization. An artificial neuron is a simplified model of a biological neuron; see Figure 1. Artificial neural systems are made up with interconnected artificial neurons.

### 4.1. Artificial Intelligence

**Figure 2:**
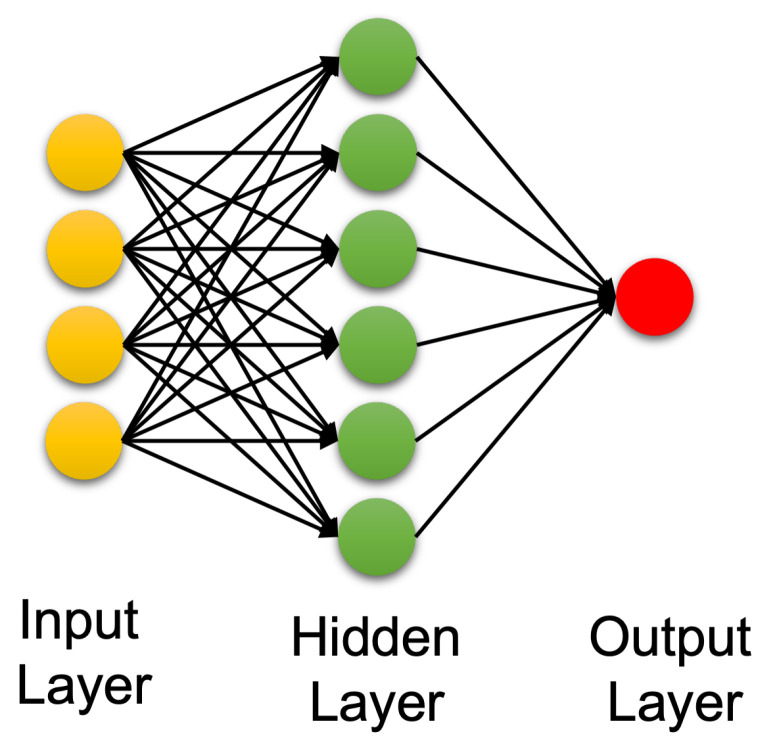
Conventional artificial neural network.

**Figure 3:**
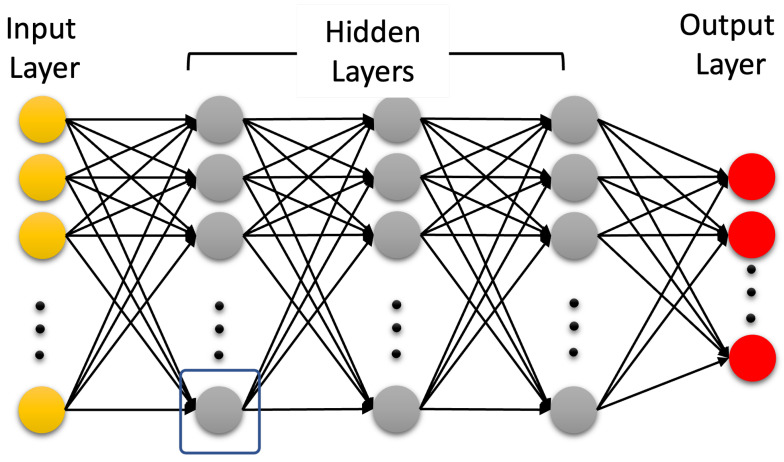
Deep learning neural network.

Artificial intelligence (AI) is a technology that attempts to make a machine an “intelligent” device. Static AI systems, known as expert systems, deal with machines that perform specific tasks. It typically uses rule-based programming and does not require any training. The limitation of expert systems is in its inability to make decisions regarding situations that are not covered by the rule base. Instead of using an expert to generate the set of rules, in machine learning, a machine learns without being programmed. It is much easier to obtain an “intelligent” machine by showing it examples of desired input and corresponding outputs than to program it manually. It uses training data to acquire knowledge that can be used for decision making. Contrary to classical expert systems, a well-trained machine learning algorithm can make a decision based on new input data that have not been seen before. In machine learning, the learning uses features extracted from the data, i.e., it does not operate on raw data directly. However, in deep learning, which is a sub field of machine learning, learning and decision making uses only raw clean data without human interference. It uses multi-layer artificial neural networks and attempts to operate in a similar manner to the brain. A conventional neural network is made up of an input layer, one hidden layer, and one output layer. A deep learning neural network is a neural network with multiple hidden layers; see Figure 2 and Figure 3, respectively.

### 4.2. Machine Learning for TBI Detection

Machine learning is a data-driven algorithm that learns and update its learning as new information is provided. There are three major types of machine learning: supervised, unsupervised, and reinforcement learning machines. Supervised learning uses input and desired output data, also called labeled data, to develop a predictive model for classification and regression. The learning algorithms include regression analysis, support vector machines, naïve Bayes, and decision tree. Unsupervised training or learning without a teacher uses only input data for the purpose of identifying patterns/structures in the data or clustering. A well-known learning algorithm is the K-means algorithm. Reinforcement learning ML is not provided with any data. It is provided with only a response to tell whether the output is true or false. Reinforcement learning is typically used for the brain–computer interface (BCI) [41,42,43,44,45,46,47,48,49]. The recent growth in data in different application areas has led to the availability of a huge amount of data, known as big data. The best example of big data availability is in the healthcare industry. To take advantage of such data, machine learning was one of the artificial intelligence technologies used [48,50,51]. This section focuses on the application of machine learning to TBI detection.

At the high level, the ML TBI detection based approach uses features extracted from the EEG(qEEG) data, as is the case for the conventional approach, then uses a learning algorithm for detection/classification. Supervised learning ML is used for classification, such as the presence or absence of a TBI in EEG signals. Supervised ML using support vector machine for TBI detection was done in [52,53], among others. The performance of the decision tree (DT), random forest (RF), and K-nearest neighbors (KNN) supervised learning algorithms was compared in [54] using random sampling and independent validation, respectively. In the study of [55], carried out using a relatively small sample of patients, the DT and KNN performed the worst. The study showed several interesting facts. First, the performance depends on the feature selection used. The second and most surprising one is that the performance using raw data and "artifacts free" data was similar. This appears to be a good indicator that existing artifact removal techniques are not helpful. A ML survey paper was published in 2020 [56]. The survey listed the 14 qEEG features used by different researchers and pointed out that the relative and absolute band powers, total EEG power of all frequency bands between 0.5–20 Hz, EEG-reactivity (EEG-R) and channels coherence were the most common features used in a decreasing order. Given the previous observation about artifact removal, one would question the sensitivity of the qEEG related to signal powers. A brain wave in a EEG signal is much weaker than the background signal and noise, resulting in a low signal-to-noise ratio (SNR). This is because when artifacts and noise are not completely or significantly removed, the EEG signal powers will be significantly affected by the powers due to artifacts in brain wavebands. Various ML learning algorithms were used with different qEEG features. The survey [56] revealed that there are no universal qEEG features that can be used to truly compare the performance of the different supervised ML algorithms using the same significant training data set. As pointed out in [57], ML algorithms do not perform any better in the prognosis of TBI than classical regression methods. In [54], the authors presented a review of wearable technologies and machine learning methodologies for the systematic detection of mild traumatic brain injuries. They concluded that more research is needed to develop a reliable and sensitive platform to diagnose mTBI, especially in its acute phase. Another area of AI, known as deep learning (DL) that can operate directly on raw data and does not need human intervention to extract features, is discussed next.

### 4.3. Artificial Neural Networks: Deep Learning Neural Networks

**Figure 4:**
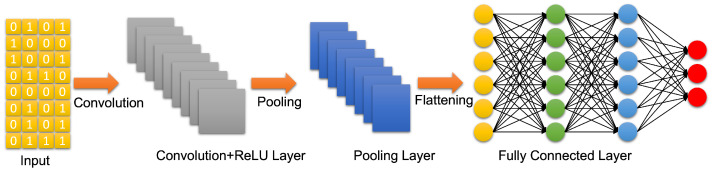
Convolutional neural network (CNN).

**Figure 5:**
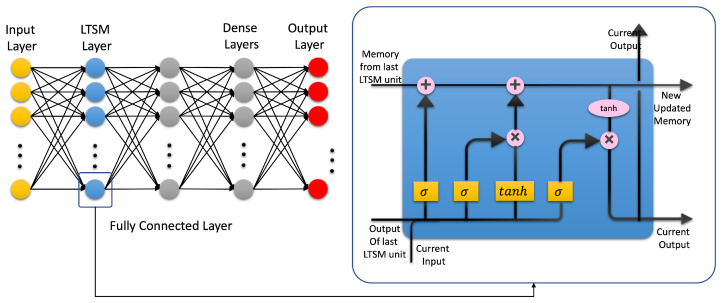
Recurrent neural network (RNN).

**Figure 6:**
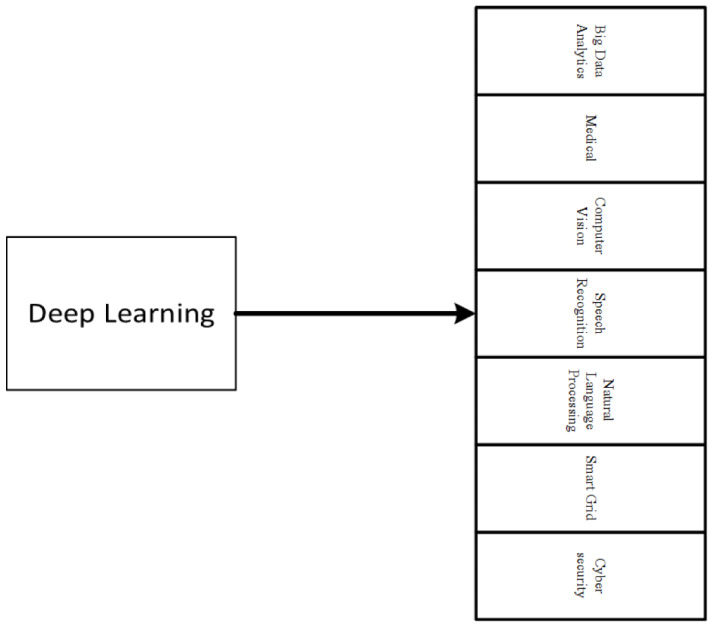
Deep learning applications.

A deep learning neural network is an artificial neural network with more than two hidden layers. In the open literature, there seem to be confusion between ML and DL. DL is a subfield of ML that uses artificial neural networks and attempts to operate in a similar way as the brain in terms of learning and generalization. There are three types of deep learning neural networks: artificial neural networks (ANN), convolutional neural networks (CNN), and recurrent neural networks (RNN). They are shown in Figure 3, Figure 4 and Figure 5, respectively. Contrary to ML, a deep learning neural network can operate directly on raw data instead of needing human intervention to select features extracted from the data whenever needed. Furthermore, DL is used for establishing complex maps between the inputs and the outputs. Typically, DL requires a high performance computing platform and a large amount of data for learning. The computing platform needs graphical and tensor processing units to reduce the processing time. DL has been applied to big data [58], medical [59,60], computer vision [61,62], power systems [63], nuclear power [64], etc. (Figure 6). As an example, ANN has been applied to detect TBI using CT scans of children admitted to the emergency unit [65]. They used CT scans to train an ANN to decide whether a patient is a clinically relevant TBI (CRTBI). CRTBI features are defined by the multicenter Pediatric Emergency Care Applied Research Network (PECARN). According to [65], this study gave excellent results. However, as discussed before, CT scans can only detect structural brain damage. It will be interesting if the study focuses on the detection of mTBI.

Even though DL has been successfully used in many areas, relatively, it has not been used as much when it comes to TBI detection using EEG. As pointed out in [66], even though the number of publication using EEG and DL is exponentially increasing, the average number of papers that use EEG in different applications is only about 50 in 2020. DL was used to detect seizures using EEG [66,67,68]. In [67], signal-to-image conversion was used to convert the EEG signal to a time–frequency image. Two conversion techniques were used: the short-term Fourier transform (STFT) and continuous wavelet transform (CWT) applied to different segments of the EEG signal of length 1.47 s each. The study showed that the CWT-based technique outperformed the STFT.

## 5. Future of TBI Detection

Even though brain waves contain very useful dynamical information capable of shedding lights on traumatic brain injury, especially at the time of the injury, the brain waves information is clouded by noises and artifacts in the collected EEG signals. As discussed previously, the current artifact removal techniques make unrealistic assumptions regarding the stationarity of the brain waves and the fact that artifacts linearly add to the brain waves in the EEG signals. These assumptions can significantly impact the quality of the extracted features from EEG signals, i.e., qEEG. For these reasons, the current state of technology of EEG based TBI detection cannot reach its full potential regardless of whether classical or artificial intelligence techniques are used. Currently, clinical EEG tests that attempt to remove some artifacts before collecting EEG data, may not reveal mTBI with a high confidence level. Nevertheless, the dynamical nature of the brain waves at the time of the brain injury may have the best chance of detecting mTBI once the signal-to-noise ratio is increased and artifacts are properly removed. For this reason, the following recommendations are proposed.

### 5.1. Multi Channel EEG Data Collection

Brain waves already provide good temporal resolution. For improved brain mapping resolution using EEG signals, multiple electrodes (channels) distributed in a strategic way across the brain are needed. In current clinical practice, typically 20 to 25 EEG channels are used. This number needs to increase. A study that determines the optimal number of channels and the strategic location of the electrodes for best spatial mapping needs to be carried out. The development of new electrodes that improve the contact with the brain that are insusceptible to patient head movement are needed for better quality brain waves. It is obvious that as the number of electrodes (channels) increases, the computational requirement for real time acquisition and processing increases. A study of the requirement of the hardware platform specifications, especially for a standalone portable EEG based detection system, is needed.

### 5.2. EEG Preprocessing

Given the nonlinear and nonstationary nature of a brain wave, proper filtering and artifact removal should be applied while taking into consideration the signal nature. A good start is the book [69] and the technique called time scale decomposition (TSD) presented in [70]. For artifact removal, one possible approach is to use deep learning to cluster the different artifacts in the EEG signal, then filter them out before TBI detection takes place. To improve the quality of artifact removal using the deep learning approach, large sets of data are needed. For this purpose, it is highly desirable to establish a worldwide data bank of EEG signals, using the same format, along with the clinical diagnosis.

### 5.3. Universal qEEG for TBI Detection and Need for Universal Testing

Currently, there are many EEG features used for TBI detection with different performance measures tested on small data sets. Further research is needed to determine the optimum number of independent qEEG for TBI/mTBI detection and establish a metric that can be used by all clinical studies. One possible universal feature is the correlation matrix of neighboring channels signals [71] after artifact removal. After proper preprocessing, Deep artificial neural network has a great potential for detection/prognosis using artifacts free EEG data collected from different data bases as previously discussed. Additional features/information provided by the surgeon/expert may be added as additional input to the DL for improved performance.

## 6. Conclusions

This paper has provided a brief review of the history of EEG, the techniques currently used for TBI detection and their limitations. Neuroimaging-based techniques can provide accurate structural mapping of the brain as well as functional mapping. However, the time resolution of neuroimaging information is rather poor. In situations where time resolution is of the essence, as is the case of TBI, capturing the brain wave’s dynamical behavior in the first two minutes after injury is crucial for mTBI detection. Besides its portability and low cost, EEG can provide the needed time resolution. However, in the current state of the art of TBI detection using current techniques, this paper has pointed out that current TBI detection using EEG cannot reach its full potential and will likely miss detecting mTBIs. Nevertheless, brain waves contain valuable information that can be used for many applications besides mTBI deletion and monitoring. Further research work is recommended to deal with the issues of extracting the brain waves from the EEG signals. It is concluded that EEG is an economical and portable technology that provides useful information about a TBI as soon as it happens. Furthermore, EEG testing can complement neuroimaging technologies for improved TBI detection and localization. However, it was found that in its current state of the art, the conventional mathematically based EEG prepossessing and quality EEG (qEEG) have ways to go from the engineering side as well as the clinical side to use EEG as a reliable tool for TBI diagnosis with a minimum false rate. Then the paper focused on a relatively new approach that uses artificial intelligence. Benefits and limitations were discussed with some details. Finally, the paper suggested new research directions to overcome some of the challenges on the recording as well as the processing, feature extraction, and detection sides that face the use of EEG-based technology for TBI detection and long-term continuous monitoring of the brain activities.

## Data Availability

Not applicable.
